# Determinants of Functional Dependency and Long-Term Care Needs Among Older Mexican Adults

**DOI:** 10.3390/healthcare14030312

**Published:** 2026-01-27

**Authors:** Sandra Luz Valdez-Avila, Myo Nyein Aung, Motoyuki Yuasa

**Affiliations:** 1 Department of Global Health Research, Graduate School of Medicine, Juntendo University, Tokyo 113-8421, Japan; valdez.wv@juntendo.ac.jp (S.L.V.-A.); moyuasa@juntendo.ac.jp (M.Y.); 2 Faculty of International Liberal Arts, Juntendo University, Tokyo 113-8421, Japan; 3 Advanced Research Institute for Health Sciences, Juntendo University, Tokyo 113-8421, Japan

**Keywords:** long-term care, functional dependency, AGGIR scale, older adults, Mexico, socioeconomic factors

## Abstract

**Background:** Low and middle-income countries (LMICs) such as Mexico are experiencing rapid population aging, accompanied by increasing levels of functional dependency and growing long-term care (LTC) needs. **Objectives:** We aimed to identify the factors associated with varying levels of functional dependency in order to assist population health planning and LTC policy in aging populations in Mexico. **Methods:** This cross-sectional study analyzed data from the 2021 wave of the Mexican Health and Aging Study (MHAS). Functional dependency was assessed through a modified Autonomie Gérontologie Groupes Iso-Ressources (AGGIR) scale, adapted to incorporate cognitive and physical assessments suitable for the Mexican context. Socioeconomic, health-related, and psychological variables were examined using ordinal logistic regression models. **Results:** Among 8049 participants included in the analysis, 87.08% were classified with non-to-mild dependency, 9.13% with moderate dependency, and 3.79% with severe dependency. More severe levels of functional dependency were associated with older age, lower educational attainment, not having a partner (being single, widowed, separated or divorced), and the presence of chronic conditions such as hypertension and cardiovascular disease. **Conclusions:** In contrast, higher educational attainment and regular physical activity were associated with less severe levels of dependency. These associations highlight the multifactorial nature of dependency in later life. The application of a graded, multidimensional dependency classification provides a more comprehensive and differentiated understanding of care needs than binary functional measures. This population-level perspective may support the prioritization of healthy aging strategies and long-term care planning in rapidly aging middle-income settings such as Mexico.

## 1. Introduction

Latin American countries, including Mexico, are facing a rapid increase in their aging population [1,2]. In Mexico, the proportion of adults aged 60 years and older is projected to rise from 12.4% in 2024 to over 20% by 2040 [3]. This demographic transition poses important public health challenges, particularly regarding the prevention and management of functional dependency, in order to maximize quality of life and foster healthy aging communities [4,5].

Functional dependency—defined as limitations in the ability to perform essential Activities of Daily Living (ADLs) and Instrumental Activities of Daily Living (IADLs) [6,7]—is both an indicator and a driver of health decline, social isolation, and increasing healthcare utilization [8,9,10,11,12]. Although global research has identified multiple determinants of functional dependency [4,9,13], analyses that examine dependency across graded severity levels remain limited in the Mexican context. By adapting the AGGIR framework to data from the MHAS, this study applies a multidimensional and graded classification of functional dependency, allowing for a more differentiated characterization of care needs among older adults. Mexico’s socioeconomic conditions, healthcare system, and cultural context shape patterns of functional decline, underscoring the need for context-specific evidence to inform policy and care strategies [14,15].

This study addresses this gap by analyzing nationally representative data from the 2021 wave of the MHAS using a modified version of the AGGIR scale. The objectives are twofold: (1) to examine population-level associations between sociodemographic, health, and psychosocial factors and varying levels of functional dependency among older Mexican adults; (2) to generate evidence relevant for informing long-term care policies and care models aimed at reducing dependency and supporting healthy aging in Mexico.

## 2. Materials and Methods

### 2.1. Study Design and Sample

This is a cross-sectional study conducted using data from the 2021 wave of the MHAS, a nationally representative survey of adults aged 50 years and older [16]. The analytical sample included community-dwelling adults aged 60 years and older. The age cutoff was selected to align with the age threshold commonly used in public policy frameworks in Mexico for the allocation of social and health-related resources, including long-term care planning. This restriction also ensures conceptual consistency with the adapted AGGIR-based dependency classification, which was originally developed to assess functional dependency and long-term care needs among older adults.

From the initial sample (*n* = 17,788), participants younger than 60 years were excluded, resulting in a sample of 10,482 individuals. Participants with missing information on any functional, cognitive, ADL or IADL items required to operationalize the modified AGGIR scale were excluded (*n* = 9238). Additional exclusions due to missing covariate data accounted for 1189 participants, resulting in a final analytic sample of 8049 individuals. A complete-case approach was adopted, as imputing core classification domains could introduce substantial misclassification.

### 2.2. Assessment of Functional Dependency

Functional dependency was assessed using a modified version of the AGGIR scale, a classification system widely used in France to determine long-term care eligibility and intensity. The original AGGIR framework distinguishes between core indicators of autonomy—such as coherence, orientation, communication, transfers, displacement, dressing, eating, elimination—and illustrative variables that provide contextual information [17,18].

The modified AGGIR classification was developed using a conceptual and methodological framework aimed at translating a clinically grounded dependency scale into a population-based operationalization suitable for secondary survey data. A domain-based mapping strategy was employed to ensure conceptual equivalence between the original AGGIR framework and available MHAS indicators, integrating cognitive function, locomotor autonomy, and limitations in ADLs and IADLs [17,19]. Items requiring continuous clinical observation or institutional documentation—central to the original AGGIR scoring—were not available in MHAS and were therefore excluded. This decision prioritized conceptual equivalence over item-level replication, consistent with established approaches in population health research [4,20].

Each AGGIR level (GIR 1–GIR 6) was defined using explicit and reproducible criteria based on combinations of cognitive performance, confinement to bed, locomotor limitations, and ADL/IADL difficulties (Table 1). Participants were assigned to the most severe level of functional dependency for which they met all criteria, following the hierarchical logic of the original AGGIR system, where lower-numbered GIR categories reflect greater dependency severity. For analytical purposes, the six GIR levels were collapsed into three ordered dependency categories: no dependency (GIR 5–6), mild–moderate dependency (GIR 3–4), and severe dependency (GIR 1–2).

The construct validity of the modified classification is supported by the consistency and plausibility of its associations with well-established correlates of functional dependency reported in the literature, including advanced age, chronic disease burden, depression, and physical limitations [20,21]. Internal consistency was assessed using Cronbach’s alpha (α = 0.58) and interpreted within the conceptual framework of AGGIR as a multidimensional, formative classification system. In such indices, modest alpha values are theoretically expected and do not necessarily indicate poor measurement quality [22,23]. To further assess statistical coherence, multicollinearity was evaluated using the variance inflation factor (VIF). In the final model, mean VIF was 1.12, indicating minimal redundancy among domains [24,25].

### 2.3. Covariates

A comprehensive set of sociodemographic, health-related, and psychological variables was selected based on established literature linking social, behavioral, and clinical factors with functional decline in older adults [26,27]. Age was treated as a continuous variable for descriptive analyses and categorized into five-year groups (60–65, 66–70, 71–75, 76–80, and >80 years) for analyses of dependency distribution and regression modeling. Educational attainment was defined as the highest level of schooling completed (no formal education, primary school, junior high school, and high school or higher). Marital status was categorized as single, married or in a civil union, or divorced, separated or widowed.

Chronic conditions—including diabetes, hypertension, cancer, asthma, emphysema, arthritis and stroke—were assessed using self-reported physician diagnoses (coded as 0 = no, 1 = yes). Body mass index (BMI) was categorized according to World Health Organization (WHO) criteria (<18.5: underweight, 18.5–24.9: normal, 25–29.9: pre-obesity, 30–34.9: obesity class I, 35–39.9: obesity class II, >40: obesity class III). Falls, fractures, loss of appetite, exhaustion, and urinary incontinence (urge or effort) during the previous two years were also assessed using single-item questions (coded as 0 = no, 1 = yes).

Depressive symptoms were measured using a nine-item depression screening questionnaire, with a cutoff point of ≥5 indicating depression [28]. Physical activity was defined as engaging in exercise three or more times per week during the previous two years (0 = no, 1 = yes). Alcohol use (never, former, moderate/heavy based on weekly intake guidelines) and tobacco use (never, former, current) were defined according to established criteria.

### 2.4. Statistical Analysis

Descriptive statistics summarized participant characteristics across dependency levels. Group differences were evaluated using chi-square tests for categorical variables and analysis of variance (ANOVA) for continuous variables. Associations between explanatory factors and functional dependency were examined using ordinal logistic regression models. The proportional odds assumption was assessed by comparing ordinal and multinomial logistic models; the final model satisfied this assumption.

Variables with a *p*-value < 0.20 in bivariate analysis, along with conceptually relevant variables, were included in the multivariable model to account for potential confounding. Results are presented as adjusted odds ratios (aOR) with 95% confidence intervals. Multicollinearity was assessed using variance inflation factors with values < 2 indicating acceptable levels. All analyses were performed using Stata SE version 17.

### 2.5. Sensitivity Analysis

To assess the robustness of the complete case approach and explore the potential impact of exclusions due to missing data, a sensitivity analysis was conducted. Ordinal logistic regression models were re-estimated including participants who were initially excluded from the main analysis due to missing covariate information but had sufficient data to classify functional dependency (9238). The direction and magnitude of associations for key predictors remained consistent with the main findings, supporting the robustness of the results.

## 3. Results

### 3.1. Sociodemographic Characteristics

A total of 8049 community-dwelling Mexican adults aged 60 years and older from the 2021 wave of the MHAS were included in the analytic sample. According to the modified AGGIR classification, 87.08% of participants were classified as having no to mild dependency, 9.13% as having moderate dependency, and 3.79% as having severe dependency. The mean age of the sample was 70.99 years (SD 7.72). Women accounted for 54.26% of participants; 63.65% had primary education or less; and 62.01% were married or living in a civil union.

Sociodemographic characteristics stratified by dependency level are presented in Table 2. Higher levels of functional dependency were more frequently observed among older age groups, particularly among individuals aged 80 years and older. Dependency severity also varied by educational attainment. Participants with primary education or less constituted a substantially larger proportion of the severe dependency group compared with those classified as having no-to-mild dependency.

Differences were also observed in partnership status. Individuals without a partner (single, widowed, separated, or divorced) represented a higher proportion of the moderate and severe dependency categories compared with the no-to-mild dependency group.

Health-related, psychological, and lifestyle characteristics across dependency levels were examined using bivariate analyses and are presented in Supplementary Tables S1 and S2. Overall, higher dependency levels were accompanied by a greater prevalence of chronic conditions, functional limitations, and psychosocial vulnerabilities. In contrast, engagement in regular physical activity was less frequent among individuals classified in higher dependency categories.

### 3.2. Multivariable Regression Analysis

Results from the ordinal logistic regression analysis are presented in Table 3. After adjustment for sociodemographic, health-related, psychological, and lifestyle variables, advanced age was strongly associated with higher levels of functional dependency. Compared with adults aged 60–69 years, individuals aged 80 years and older had more than twice the odds of being classified in a more severe dependency category (adjusted odds ratio [aOR] 2.38, 95% CI 1.88–3.02).

Educational attainment showed a clear inverse association with dependency severity. Participants with high school education or higher had lower odds of being classified in higher dependency categories compared with those with primary education or less (aOR 0.56, 95% CI 0.42–0.75). Partnership status remained significantly associated with dependency, with individuals without a partner exhibiting higher odds of more severe dependency levels.

Several chronic health conditions were associated with more severe dependency levels. A history of stroke (aOR 2.07, 95% CI: 1.47–2.91), hypertension (aOR 1.19, 95% CI: 1.02–1.39) and diabetes (aOR 1.38, 95% CI: 1.19–1.61) was associated with increased odds of greater dependency severity. Indicators of psychosocial vulnerability were also relevant: depression (aOR 1.70, 95% CI: 1.44–1.99) and frequent severe fatigue or exhaustion (aOR 1.63, 95% CI: 1.38–1.94) were associated with more severe dependency categories.

In contrast, regular physical activity was associated with lower odds of being classified in higher dependency categories (aOR 0.65, 95% CI 0.54–0.78). Moderate or heavy alcohol consumption was also inversely associated with dependency severity (aOR 0.63, 95% CI 0.48–0.84).

## 4. Discussion

This study provides updated national estimates of functional dependency among older Mexican adults and examines socioeconomic, psychological, and health-related factors associated with varying levels of dependency. The observed patterns are consistent with international evidence indicating that advanced age, multimorbidity, and social vulnerability are associated with more severe levels of dependency in aging populations. These findings align with a life-course perspective, which conceptualizes functional dependency as the cumulative result of health and social disadvantages accrued over time rather than as an inevitable consequence of aging [29,30].

Several health-related conditions were associated with greater dependency severity. In particular, stroke and diabetes demonstrated robust associations with higher dependency levels (Table 3), underscoring the importance of chronic disease management and access to rehabilitation services within LTC and health system planning frameworks [31,32]. Depressive symptoms were also consistently associated with more severe dependency, highlighting the close interrelationship between mental health and functional status in later life and supporting the integration of psychosocial assessment within primary and geriatric care models [33,34].

Socioeconomic characteristics exhibited clear gradients across dependency levels. Higher educational attainment was consistently associated with lower dependency severity, in line with evidence linking education to health literacy, access to care, and adaptative health behaviors across the life course [13,35,36]. Partnership status also showed a clear association with dependency gradients, reflecting the role of social support, shared resources, and informal caregiving in maintaining functional capacity in later life [37,38,39].

The inverse association between moderate alcohol consumption and dependency should be interpreted with caution. Similar counterintuitive findings have been reported in observational studies of aging populations and may reflect survival bias, reverse causality, or differential reporting by health status rather than a true protective effect [40,41,42].

Regular physical activity was associated with lower dependency levels, reinforcing evidence that physical activity contributes to physical resilience and delayed functional decline among older adults [43,44].

In the context of increasing life expectancy and a growing burden of chronic conditions, the population burden of functional dependency is expected to rise substantially in the coming decades [31,45,46]. Strengthening coordinated and formalized LTC systems that integrate health, social, and family-based support will therefore be essential to respond effectively to the needs of rapidly aging populations such as Mexico.

### Strengths and Limitations

A key contribution of this study lies in the operationalization of a modified AGGIR-based dependency classification within a large, nationally representative survey of older adults in Mexico. This study demonstrates the feasibility and conceptual relevance of translating a clinically grounded dependency framework into a survey-based operationalization suitable for population health surveillance.

Several limitations should be acknowledged. The cross-sectional design precludes causal inference, and all associations should be interpreted as correlational. Reliance on self-reported information may introduce recall or reporting bias, particularly for chronic conditions and functional limitations. While MHAS is nationally representative, contextual differences may limit generalizability [4,30].

The use of a complete-case approach may introduce the potential for selection bias. To assess the robustness of the findings, an additional sensitivity analysis was conducted, re-estimating the models with participants previously excluded due to missing covariate information. The consistency of the observed associations across analyses supports the stability of the main results.

The modified AGGIR scale represents a multidimensional classification of functional dependency, which enhances conceptual relevance but may introduce some imprecision between adjacent dependency levels. The observed Cronbach’s alpha (α = 0.58) reflects the heterogeneous and formative nature of the construct. Low variance inflation factors support the internal coherence of the classification.

## 5. Conclusions

This study applied a multidimensional, graded adaptation of the AGGIR framework to nationally representative data from the MHAS to examine functional dependency among older adults in Mexico. By integrating cognitive, functional, and locomotor domains into an ordinal dependency classification, the study addressed its objective of moving beyond conventional binary ADL/IADL measures and provided a population-level characterization of dependency severity.

Profound level of functional dependency was associated with advanced age, lower educational attainment, partnership status, chronic health conditions, and psychosocial vulnerabilities, underscoring the multifactorial nature of dependency in later life. These patterns highlight the utility of the adapted classification for identifying population groups with greater potential care needs.

We expected that it would be particularly relevant for health system planning and long-term care policy discussions in rapidly aging middle-income countries. The consistency of the observed associations with existing literature supports the relevance of this approach for informing strategies aimed at monitoring functional dependency and anticipating long-term care needs in Mexico’s aging population.

## Figures and Tables

**Table 1:** Construction of the modified AGGIR Classification Scale. MHAS, 2021.

Original AGGIR Level	Conceptual Definition (AGGIR)	Operational Criteria in MHAS (Key Domains)	Modified AGGIR Level (This Study)	Dependency Category (3-Level)
GIR 1	Essential presence of a caregiver; very low autonomy	Confined to bed (>30 days or unable to walk across a room) and severe cognitive impairment ^a^ (≥2 cognitive tests ≤ −2 SD for age) and difficulty in ≥1 ADL or ≥2 IADLs	GIR 1	Severe dependency
GIR 2	Low autonomy; constant supervision recommended	Confined to bed or severe cognitive impairment ^a^ with ADL/IADL difficulty (excluding GIR 1 cases)	GIR 2	
GIR 3	Partial locomotor autonomy; daily assistance required	Partial locomotor autonomy (difficulty walking across a room and transferring) and need help to eat or use the toilet; includes cognitively impaired without IADL difficulty	GIR 3	Mild–moderate dependency
GIR 4	Difficulty with personal care and household tasks	(a) Unable to transfer but able to walk once up, with help bathing/dressing or (b) no locomotor difficulty but needs help with eating or using the toilet	GIR 4	
GIR 5	Occasional assistance for IADLs or minor ADLs	No cognitive impairment and difficulty in ≥1 IADL OR difficulty bathing/dressing only, without other ADL limitations	GIR 5	No–mild dependency
GIR 6	Full autonomy	No cognitive impairment, not confined to bed, and no ADL/IADL limitations	GIR 6	

^a^ Cognitive domain included constructional praxis, verbal learning, visual scanning, constructional praxis recall, and delayed verbal recall. ADLs and IADLs were derived from standard MHAS items (e.g., eating, bathing, dressing, toileting, shopping, medication management, financial management).

**Table 2:** Sociodemographic characteristics by dependency level in older Mexican adults (N = 8049). MHAS, 2021.

Sociodemographic Characteristics	No/Mild Dependency		Moderate Dependency			Severe Dependency		Total	
	Values, n (%)	Mean (SD)	Values, n (%)		Mean (SD)	Values, n (%)	Mean (SD)	Values, n (%)	Mean (SD)
Dependency level	7009 (87.08)		735 (9.13)			305 (3.79)		8049	
Age (years)		70.49 (7.47)			74.76 (8.56)		73.41 (8.23)		70.99 (7.72)
60–65	2206 (31.47)		126 (17.14)			63 (20.66)		2395 (29.76)	
66–70	1454 (20.74)		111 (15.10)			53 (17.38)		1618 (20.1)	
71–75	1604 (22.88)		156 (21.22)			69 (22.62)		1829 (22.72)	
76–80	996 (14.21)		151 (20.54)			63 (20.66)		1210 (15.03)	
80 and more	749 (10.69)		191 (25.9)			57 (18.68)		997 (12.39)	
Sex		^- a^			^- a^		^- a^		^- a^
Female	3705 (52.86)		487 (66.26)			175 (57.38)		4367 (54.26)	
Education		6.62 (4.83)			4.88 (4.28)		4.73 (4.27)		6.39 (4.8)
Did not go to school	784 (11.18)		154 (20.95)			73 (23.93)		1011 (12.56)	
Primary school	3552 (50.68)		392 (53.33)			168 (55.08)		4112 (51.09)	
Secondary school	1412 (20.15)		123 (16.73)			36 (11.8)		1571 (19.52)	
High school and above	1261 (17.99)		66 (8.98)			28 (9.18)		1355 (16.83)	
Marital status		^- a^			^- a^		^- a^		^- a^
Single	287 (4.09)		33 (4.49)			13 (4.26)		333 (4.13)	
Married/civil union	4452 (63.52)		372 (50.61)			167 (54.75)		4991 (62.01)	
Divorced/separated	2270 (32.39)		330 (44.9)			125 (40.98)		2725 (33.86)	
Social security		^- a^			^- a^		^- a^		^- a^
No social security	1375 (19.62)		170 (23.13)			68 (22.3)		1613 (20.04)	
With social security	5634 (80.38)		565 (76.87)			237 (77.7)		6436 (79.96)	

^a^ Not applicable.

**Table 3:** Multivariable analysis of factors associated with dependency in older Mexican adults (N = 8049) ^a^.

Independent Variables	Multivariable Analysis, OR ^b^ (95% CI)
Age (60–65 as reference)	
66–70	1.13 ^c^ (0.90–1.42)
71–75	1.27 ^e^ (1.03–1.58)
76–80	1.81 ^d^ (1.44–2.27)
>80	2.38 ^d^ (1.88–3.02)
Sex (male as reference)	
Female	0.92 ^c^ (0.77–1.11)
Education (did not go to school as reference)	
Primary school	0.72 ^d^ (0.59–0.87)
Secondary school	0.67 ^d^ (0.53–0.86)
High school and above	0.56 ^d^ (0.42–0.75)
Poverty (Nonfood insecurity as reference)	
Food insecurity	0.99 ^c^ (0.85–1.17)
Marital status (single as reference)	
Married/civil union	0.69 ^e^ (0.49–0.98)
Divorced/separated/widow	0.78 ^c^ (0.55–1.10)
Hypertension (not being diagnosed with high blood pressure as reference)	
Diagnosis of hypertension	1.19 ^e^ (1.02–1.39)
Diabetes (not being diagnosed with diabetes as reference)	
Diagnosis of diabetes	1.38 ^d^ (1.19–1.61)
Stroke (not being diagnosed with a stroke as reference)	
Diagnosis of stroke	2.07 ^d^ (1.47–2.91)
Falls (not having suffered any falls in the last 2 years as reference)	
One or more falls in the last 2 years	1.42 ^d^ (1.23–1.65)
Fractures (any fractures in the last 2 years as reference)	
Hip fracture	3.13 ^d^ (2.04–4.81)
Fracture in other bones	1.52 ^d^ (1.17–1.96)
Alcohol intake (never used as reference)	
Currently does not drink alcohol	0.94 ^c^ (0.78–1.13)
Moderate/heavy user	0.63 ^d^ (0.48–0.84)
Smoking (never smoking as reference)	
Former smoker	1.01 ^c^ (0.85–1.20)
Current smoker	0.94 ^c^ (0.72–1.24)
Body mass index (normal weight as a reference)	
Underweight	1.25 ^c^ (0.76–2.05)
Pre obesity	1.03 ^c^ (0.87–1.22)
Obesity I	1.02 ^c^ (0.82–1.25)
Obesity II	1.40 ^e^ (1.04–1.88)
Obesity III	1.30 ^c^ (0.77–2.20)
Physical activity in the last 2 years (no physical activity as a reference)	
Activity 3 or more times per week	0.65 ^d^ (0.54–0.78)
Urinary incontinence in the last two years (not having incontinence as reference)	
Stress or urge urinary incontinence	1.44 ^d^ (1.24–1.68)
Severe fatigue or exhaustion in the last 2 years (not having fatigue as reference)	
Frequent severe fatigue or exhaustion	1.63 ^d^ (1.38–1.94)
Decreased food intake in the last two years (no decrease in intake as reference)	
Decreased food intake	1.56 ^d^ (1.34–1.82)
Recreational activities (does not perform recreational activities as reference)	
Perform activities (reading, doing puzzles, playing cards, chess, watching TV, or doing crafts).	0.76 ^d^ (0.65–0.88)
Depression (not having depression at the time of the survey)	
Depression	1.70 ^d^ (1.44–1.99)

^a^ Model parameter: pseudo *r*^2^ = 0.11, LR test of proportionality of odds = 824.17. ^b^ OR: odds ratio. ^c^ *p* < 0.02. ^d^ *p* < 0.01. ^e^ *p* < 0.05.

## Data Availability

The data presented in this study are openly available in the Mexican Health and Aging Study (MHAS) at https://www.mhasweb.org/DataProducts/CoreSurveyData.aspx (accessed on 24 October 2022).

## Supplementary Materials

The following supporting information can be downloaded at https://www.mdpi.com/article/10.3390/healthcare14030312/s1; https://www.mhasweb.org/Home/index.aspx (accessed on 29 January 2024). Core survey data 2021, Core Interview—Sections A, AA, C, D, E, PC, F, H, and I (Individual Level) and Sections G, J, K, and SA (Household Level). Supplementary Table S1: Health-related and psychological variables by dependency level in older Mexican adults (N = 8049). Supplementary Table S2: Lifestyle habits and other symptoms by dependency level in older Mexican adults (N = 8049).
