# Supplementary material for: Determinants of Functional Dependency and Long-Term Care Needs Among Older Mexican Adults
Source: Healthcare (Basel). 2026 Jan 27;14(3):312. doi: 10.3390/healthcare14030312 (PMC12896549; doi:10.3390/healthcare14030312)
Supplement: Supplementary file 1 [file healthcare-14-00312-s001.zip › healthcare-4028811-supplementary.pdf]

## Supplementary Tables

**Table S1.** Health related and psychological variables by dependency level in older Mexican adults (N=8,049). MHAS, 2021.

| Health and Psychological Variables | Dependency level | | | Total |
|---|---|---|---|---|
| | No / Mild | Moderate | Severe | |
| | Values, n (%) | | | |
| | 7009 (87.08) | 735 (9.13) | 305 (3.79) | 8049 |
| Hypertension | | | | |
| Not diagnosed | 3427 (48.89) | 243 (33.06) | 120 (39.34) | 3790 (47.09) |
| Diagnosed | 3582 (51.11) | 492 (66.94) | 185 (60.66) | 4259 (52.91) |
| Diabetes | | | | |
| Not diagnosed | 5092 (72.65) | 465 (63.27) | 170 (55.74) | 5727 (71.15) |
| Diagnosed | 1917 (27.35) | 270 (36.73) | 135 (44.26) | 2322 (28.85) |
| Cancer | | | | |
| Not diagnosed | 6816 (97.25) | 707 (96.19) | 285 (93.44) | 7808 (97.01) |
| Diagnosed | 193 (2.75) | 28 (3.81) | 20 (6.56) | 241 (2.99) |
| Asthma or emphysema | | | | |
| Not diagnosed | 6664 (95.08) | 669 (91.02) | 272 (89.18) | 7605 (94.48) |
| Diagnosed | 345 (4.92) | 66 (8.98) | 33 (10.82) | 444 (5.52) |
| Stroke | | | | |
| Not diagnosed | 6870 (98.02) | 701 (95.37) | 286 (93.77) | 7857 (97.61) |
| Diagnosed | 139 (1.98) | 34 (4.63) | 19 (6.23) | 192 (2.39) |
| Hearth attack | | | | |
| Not diagnosed | 6753 (96.35) | 677 (92.11) | 277 (90.82) | 7707 (95.75) |
| Diagnosed | 256 (3.65) | 58 (7.89) | 28 (9.18) | 342 (4.25) |
| Arthritis | | | | |
| Not diagnosed | 6215 (88.67) | 538 (73.2) | 237 (77.7) | 6990 (86.84) |
| Diagnosed | 794 (11.33) | 197 (26.8) | 68 (22.3) | 1059 (13.16) |

| | | | | |
|---|---|---|---|---|
| Infectious diseases in the last 2 years | | | | |
| Any infection | 6174 (88.09) | 601 (81.77) | 241 (79.02) | 7016 (87.17) |
| Kidney/Liver/Tuberculosis/Pneumonia | 835 (11.91) | 134 (18.23) | 64 (20.98) | 1033 (12.83) |
| Hospitalizations in the last year | | | | |
| None | 6380 (91.03) | 613 (83.4) | 189 (61.97) | 7182 (89.23) |
| One or more hospitalizations | 629 (8.97) | 122 (16.6) | 116 (38.03) | 867 (10.77) |
| Falls in the last 2 years | | | | |
| No | 4245 (60.56) | 310 (42.18) | 119 (39.02) | 4674 (58.07) |
| One or more falls | 2764 (39.44) | 425 (57.82) | 186 (60.98) | 3375 (41.93) |
| Fracture in the last 2 years | | | | |
| No | 6615 (94.38) | 656 (89.25) | 250 (81.97) | 7521 (93.44) |
| One or more fractures | 394 (5.62) | 79 (10.75) | 55 (18.03) | 528 (6.56) |
| Sight | | | | |
| Not impaired | 6595 (94.09) | 612 (83.27) | 243 (79.67) | 7450 (92.56) |
| Impaired | 414 (5.91) | 123 (16.73) | 62 (20.39) | 599 (7.44) |
| Hearing | | | | |
| Not impaired | 6760 (96.45) | 675 (91.84) | 284 (93.11) | 7719 (95.9) |
| Impaired | 249 (3.55) | 60 (8.16) | 21 (6.89) | 330 (4.1) |
| Limiting pain | | | | |
| No | 5951 (84.91) | 406 (55.24) | 198 (64.92) | 6555 (81.44) |
| Affects daily activities | 1058 (15.09) | 329 (44.76) | 107 (35.08) | 1494 (18.56) |
| High levels of stress | | | | |
| No | 6341 (90.47) | 600 (81.63) | 249 (81.64) | 7190 (89.33) |
| Yes | 668 (9.53) | 135 (18.37) | 56 (18.36) | 859 (10.67) |
| Depression | | | | |
| Not depressed | 5857 (83.56) | 438 (59.59) | 201 (65.9) | 6496 (80.71) |
| Depressed | 1152 (16.44) | 297 (40.41) | 104 (34.1) | 1553 (19.29) |
| Life satisfaction | | | | |

| | | | | |
|---|---|---|---|---|
| Unsatisfied | 386 (5.51) | 85 (11.56) | 26 (8.52) | 497 (6.17) |
| Satisfied | 6623 (94.49) | 650 (88.44) | 279 (91.48) | 7552 (93.83) |
| Feeling of loneliness | | | | |
| Not lonely | 6067 (86.56) | 536 (72.93) | 238 (78.03) | 6841 (84.99) |
| Lonely | 942 (13.44) | 199 (27.07) | 67 (21.97) | 1208 (15.01) |
| Decision making | | | | |
| A lot | 5893 (84.08) | 588 (80) | 239 (78.36) | 6720 (83.49) |
| Al little/not at all | 1116 (15.92) | 147 (20) | 66 (21.64) | 1329 (16.51) |

**Table S2.** Lifestyle habits and other symptoms by dependency level in older Mexican adults (N=8,049). MHAS, 2021.

| **Lifestyle habits and other symptoms** | **Dependency level** | | | **Total** |
|---|---|---|---|---|
| | **No dependency/Mild** | **Moderate** | **Severe** | |
| | | Values, n (%) | | |
| Smoking | | | | |
| Never | 6290 (89.74) | 684 (93.06) | 282 (92.46) | 7256 (90.15) |
| Former/current | 719 (10.26) | 51 (6.94) | 23 (7.54) | 793 (9.85) |
| Alcohol use | | | | |
| Never | 1080 (15.41) | 157 (21.36) | 51 (16.72) | 1288 (16) |
| Former | 4552 (64.95) | 498 (67.76) | 228 (74.75) | 5278 (65.57) |
| Moderate/heavy | 1377 (19.65) | 80 (10.88) | 26 (8.52) | 1483 (18.42) |
| Body Mass Index | | | | |
| Normal | 2252 (32.13) | 230 (31.29) | 106 (34.75) | 2588 (32.15) |
| Underweight | 85 (1.21) | 16 (2.18) | 8 (2.62) | 109 (1.35) |
| Pre-obesity | 2964 (42.29) | 280 (38.1) | 118 (38.69) | 3362 (41.77) |
| Obesity | 1708 (24.37) | 209 (28.44) | 73 (23.93) | 1990 (24.72) |
| Exercise or hard physical work three or more times per week | | | | |
| No | 4881 (69.64) | 618 (84.08) | 257 (84.26) | 5756 (71.51) |
| Yes | 2128 (30.36) | 117 (15.92) | 48 (15.74) | 2293 (28.49) |

| | | | | |
|---|---|---|---|---|
| Exhaustion | | | | |
| No | 6131 (87.47) | 495 (67.35) | 219 (71.8) | 6845 (85.04) |
| Yes | 878 (12.53) | 240 (32.65) | 86 (28.2) | 1204 (14.96) |
| Incontinence (urinary) | | | | |
| No incontinence | 5451 (77.77) | 423 (57.55) | 207 (67.87) | 6081 (75.55) |
| Effort/Urge incontinence | 1558 (22.23) | 312 (42.45) | 98 (32.13) | 1968 (24.45) |
| Grip strength | | | | |
| Strong | 5331 (76.06) | 337 (45.85) | 174 (57.05) | 5842 (72.58) |
| Weak | 1678 (23.94) | 398 (54.15) | 131 (42.95) | 2207 (27.42) |
| Eat less (last three months) | | | | |
| No | 5459 (77.89) | 419 (57.01) | 170 (55.74) | 6048 (75.14) |
| Loss of appetite or digestive problems | 1550 (22.11) | 316 (42.99) | 135 (44.26) | 2001 (24.86) |
